# Dose-Dependent Change in Elimination Kinetics of Ethanol due to Shift of Dominant Metabolizing Enzyme from ADH 1 (Class I) to ADH 3 (Class III) in Mouse

**DOI:** 10.1155/2012/408190

**Published:** 2011-11-22

**Authors:** Takeshi Haseba, Kouji Kameyama, Keiko Mashimo, Youkichi Ohno

**Affiliations:** ^1^Department of Legal Medicine, Nippon Medical School, 1-1-5 Sendagi, Bunkyo-ku, Tokyo 113-8602, Japan; ^2^Department of Pathology, Nippon Medical School, 1-1-5 Sendagi, Bunkyo-ku, Tokyo 113-8602, Japan

## Abstract

ADH 1 and ADH 3 are major two ADH isozymes in the liver, which participate in systemic alcohol metabolism, mainly distributing in parenchymal and in sinusoidal endothelial cells of the liver, respectively. We investigated how these two ADHs contribute to the elimination kinetics of blood ethanol by administering ethanol to mice at various doses, and by measuring liver ADH activity and liver contents of both ADHs. The normalized AUC (AUC/dose) showed a concave increase with an increase in ethanol dose, inversely correlating with *β*. CL_*T*_ (dose/AUC) linearly correlated with liver ADH activity and also with both the ADH-1 and -3 contents (mg/kg B.W.). When ADH-1 activity was calculated by multiplying ADH-1 content by its *V*_max⁡_/mg (4.0) and normalized by the ratio of liver ADH activity of each ethanol dose to that of the control, the theoretical ADH-1 activity decreased dose-dependently, correlating with *β*. On the other hand, the theoretical ADH-3 activity, which was calculated by subtracting ADH-1 activity from liver ADH activity and normalized, increased dose-dependently, correlating with the normalized AUC. These results suggested that the elimination kinetics of blood ethanol in mice was dose-dependently changed, accompanied by a shift of the dominant metabolizing enzyme from ADH 1 to ADH 3.

## 1. Introduction

Alcohol dehydrogenase (ADH; EC 1.1.1.1) in the liver is generally accepted to be the primary enzyme responsible for ethanol metabolism. This is supported by evidence that the level of liver ADH activity is closely correlated with the rate of ethanol metabolism [1–3] and that the metabolism *in vivo *is markedly depressed in animals treated with pyrazoles of ADH inhibitors [4, 5] and in ones genetically lacking ADH [6]. The process by which blood ethanol is eliminated was traditionally assumed to follow zero-order [7] or single Michaelis-Menten (M-M) kinetics [8, 9], even though mammalian livers actually contain three kinds of ADH isozymes (Class I, II, III) with different *K*_*m*_s for ethanol [10, 11]. Thus, it was commonly thought that the elimination process was regulated by Class I ADH (ADH 1), which distributes mainly in parenchymal liver cells [12], because this classically known ADH has the lowest *K*_*m*_ among the three liver ADH isozymes and because its activity saturates at millimolar levels of ethanol. Indeed, mice genetically lacking ADH 1 have been used to demonstrate that ADH 1 is a key enzyme in systemic ethanol metabolism [13, 14]. However, studies on these ADH-1-deficient animals have also shown that ethanol metabolism *in vivo* cannot be explained solely by ADH 1 [13, 14]. Although the microsomal ethanol oxidizing system (MEOS) including CYP2E1 as a main component, and catalase have been discussed for many years as candidates for non-ADH 1 pathways [15, 16], these studies have failed to clarify their roles in ethanol metabolism in mice genetically lacking these enzymes [17–19]. Moreover, the process of the elimination of blood ethanol has been shown to involve first-order kinetics [20–23], suggesting that alcohol-metabolizing enzymes with a very high *K*_*m*_ participate in systemic ethanol metabolism. ADH 3 (Class III), another major ADH, which distributes mainly in sinusoidal endothelial cells of the liver [12], has very high *K*_*m*_ for ethanol. Therefore, it shows very little activity when assayed by the conventional method with millimolar levels of ethanol as a substrate; but its activity increases up to the molar level of ethanol [10, 24]. Additionally, this ADH has been demonstrated to be markedly activated under hydrophobic conditions, which lower its *K*_*m*_ [14, 25]. Previously, liver ADH activity was assumed to be attributable solely to ADH 1 because it was responsible for most of the activity due to its low *K*_*m*_ [10, 24]. However, we have used ethanol-treated mice to show that liver ADH activity assayed by the conventional method depends not only on ADH 1 but also on ADH 3 and governs the elimination rate of blood ethanol [3]. Moreover, we have recently demonstrated using *Adh3-*null mice that ADH 3 participates in systemic ethanol metabolism dose-dependently [14].

These data suggest that systemic ethanol metabolism in mice involves both liver ADH 1 and ADH 3, possibly through the regulation of their contents and/or enzymatic kinetics. However, how these two ADH isozymes contribute to the elimination kinetics of ethanol is largely unknown.

In the present study, we investigated how these two liver ADHs contribute to the elimination kinetics of ethanol in mice by statistically analyzing the pharmacokinetic parameters of blood ethanol and the enzymatic parameters of ADH, based on a two-ADH model that ascribes liver ADH activity to both ADH 1 and ADH 3.

## 2. Methods

### 2.1. Measurement of Pharmacokinetic Parameters of Blood Ethanol

As previously described [3], male ddY mice (9 weeks old) were injected with ethanol (i.p.) at a dose of 1, 2, 3, 4.5, or 5 g/kg body weight, while the control mice were injected with saline (0 g/kg). For each dose, blood samples were taken from the tails of mice (*n* = 3) at scheduled times (0.5, 1, 2, 4, 8, and 12 h) after ethanol administration.

Blood ethanol concentration was measured with a head-space gas chromatograph [3]. The rate of ethanol elimination from blood was expressed as a *β*-value (mmol/L/h), which was calculated from a regression line fitted to the blood ethanol concentrations at various times by the linear least-squares method [26]. The area under the blood concentration-time curve (AUC) was calculated by trapezoidal integration using the extrapolation of time course curves to obtain the normalized AUC (AUC/dose) and body clearance of ethanol (CL_*T*_: the reciprocal of the normalized AUC) [23].

All animals received humane care in compliance with our institutional guidelines “The Regulations on Animal Experimentation of Nippon Medical School,” which was based on “The Guidelines of the International Committee on Laboratory Animals 1974”.

### 2.2. Measurement of Liver ADH Parameters

In order to obtain liver samples, mice were sacrificed by cervical dislocation at scheduled times during ethanol metabolism at each dose (0.5, 1, and 2 h for 1 and 2 g/kg; 0.5, 1, 2, 4, and 8 h for 3 g/kg; 0.5, 1, 2, 4, 8, and 12 h for 0, 4.5, and 5 g/kg) (*n* = 3 at each time in each dose). Each liver was homogenized in 6 vol (w/v) of extraction buffer (0.5 mM NAD, 0.65 mM DTT/5 mM Tris-HCL, pH 8.5) and centrifuged at 105,000 ×g for 1 h to obtain a liver extract.

ADH activity was measured at pH 10.7 by the conventional assay with 15 mM ethanol as a substrate, using liver extract during the times of ethanol metabolism at each dose. The ADH 1 and ADH 3 contents of liver were measured by EIA using isozyme-specific antibodies on the same samples as those used for ADH activity [3], excluding the samples at doses of 2 and 4.5 g/kg. The ADH activity and content of liver were expressed in terms of liver weight/kg body weight because these units are not influenced by hepatomegaly or variations in the total liver weight with respect to body weight. These liver ADH parameters were averaged over the ethanol-metabolizing time for each dose of ethanol and termed the liver ADH activity, the liver ADH 1 content, and the liver ADH 3 content.

The apparent *K*_*m*_ and *V*_max_ of ADH activity were determined from a Lineweaver-Burk plot with ethanol (0.1–100 mM) as a substrate, using liver extracts obtained at 1 and 4 h after ethanol administration for all doses (*n* = 3 at each time in each dose). *V*_max_ is expressed in units/mg of the sum of the ADH 1 and ADH 3 contents.

### 2.3. Two-ADH-Complex Model of Liver ADH Activity

The two-ADH-complex model, which ascribes liver ADH activity to both ADH 1 and ADH 3, is described by the function [*y* (ADH activity) = *f* (ADH 1 activity, ADH 1 content, ADH 3 activity, ADH 3 content)] for each liver extract. The *V*_max⁡_ of ADH 1 in liver extract is assumed to be a constant 4.0 units/mg, regardless of ethanol dose, because purified mouse ADH 1 usually exhibits a relatively constant *V*_max⁡_ of around 4.0 units/mg, a value that was obtained with around 15 mM ethanol as a substrate at pH 10.7 [3]. In the complex model, therefore, ADH 1 activity was calculated from [ADH 1 content × 4.0], while ADH 3 activity was assumed to be [ADH  activity − ADH  1  activity] in each liver. These assumptions are based on two facts: (1) ADH 2 (the third ADH isozyme in liver) is only responsible for a very small portion of total ADH activity in mice liver (<3%) [3], and (2) ADH 3 is activated depending on the conditions of medium [14, 25]. The calculated ADH 1 and ADH 3 activities were then averaged over the ethanol-metabolizing time for each dose of ethanol and normalized by the ratio of the average liver ADH activity of each ethanol group to that of the control. These normalized ADH activities were termed the theoretical ADH 1 and ADH 3 activities. These parameters were used for statistical analyses and correlation studies.

## 3. Results

### 3.1. Effect of Dose on Pharmacokinetics of Blood Ethanol

**Figure 1:**
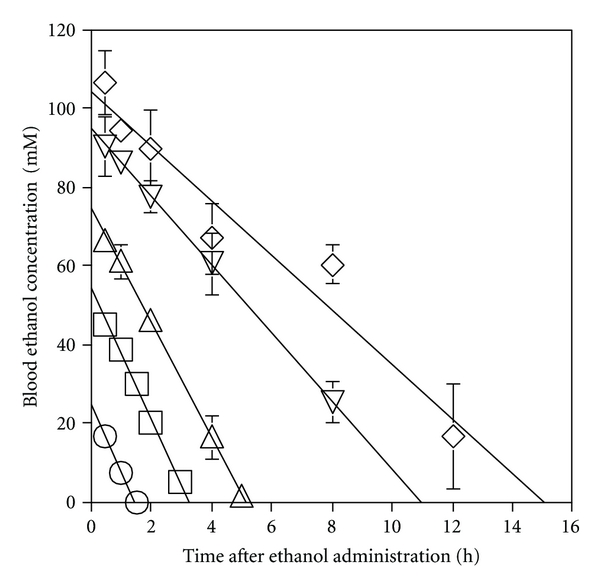
Time course of blood ethanol concentration in mice after ethanol administration (i.p.) at various doses. Each plot represents the mean ± SD of 3 mice. ○ 1 g/kg; □ 2 g/kg; *▵* 3 g/kg; ▿ 4.5 g/kg; *⋄* 5 g/kg.

**Figure 2:**
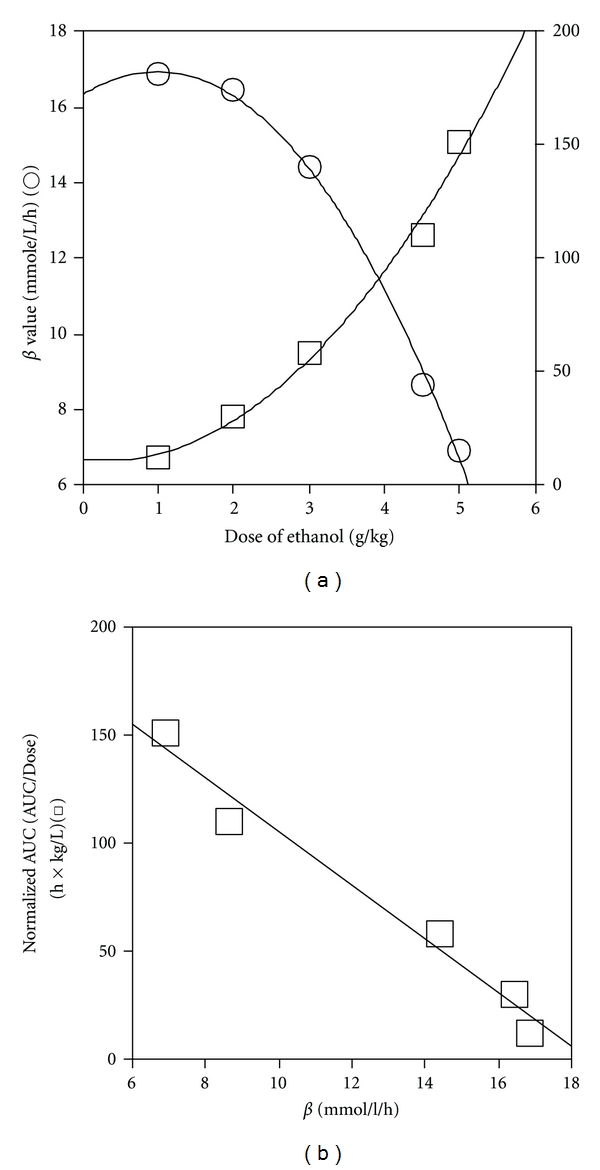
(a) Effect of ethanol dose on elimination rate (*β*) and normalized AUC (AUC/dose) of blood ethanol. (b) Correlation of normalized AUC with **β** in mice for various doses of ethanol. *β* (○) and normalized AUC (□) were calculated from the regression line fitted to the blood ethanol concentrations at each dose in Figure 1.

Figure 1 shows the time course of blood ethanol concentration in mice after the administration of ethanol at various doses. Blood ethanol elimination roughly followed zero-order or M-M kinetics, reaching a constant *V*_max_ at every dose of ethanol, as shown by the regression lines fitted to the blood ethanol concentrations at various times (*r* ^2^ = 0.996, 0.996, 0.999, 1.000, and 0.945 for doses of 1, 2, 3, 4.5, and 5 g/kg, resp.). The *β* values were 16.9, 16.5, 14.5, 8.7, and 6.9 mmol/L/h and the blood ethanol concentrations extrapolated to a time of zero (*C*_0_) were 25.2, 54.1, 74.8, 94.9, and 104.2 mM for doses of 1, 2, 3, 4.5, and 5 g/kg, respectively. The *β* values were almost constant at low doses (1 and 2 g/kg) but decreased when the dose exceeded 2 g/kg (*r* ^2^ = 0.997) (Figure 2(a)). On the other hand, the normalized AUC (AUC/dose), which negatively correlated with *β* (*r* ^2^ = 0.974) (Figure 2(b)), showed a concave increase with dose (*r* ^2^ = 0.991) (Figure 2(a)) and, therefore, exhibited a linear correlation with the square of the dose (*r* ^2^ = 0.993) (data not shown). The CL_*T*_ of ethanol, that is, the reciprocal of the normalized AUC, decreased dose-dependently along a concave curve (data not shown). This differed from the behavior of *β*, which exhibited a convex decrease.

### 3.2. Effect of Ethanol Dose on Liver ADH Parameters

**Figure 3:**
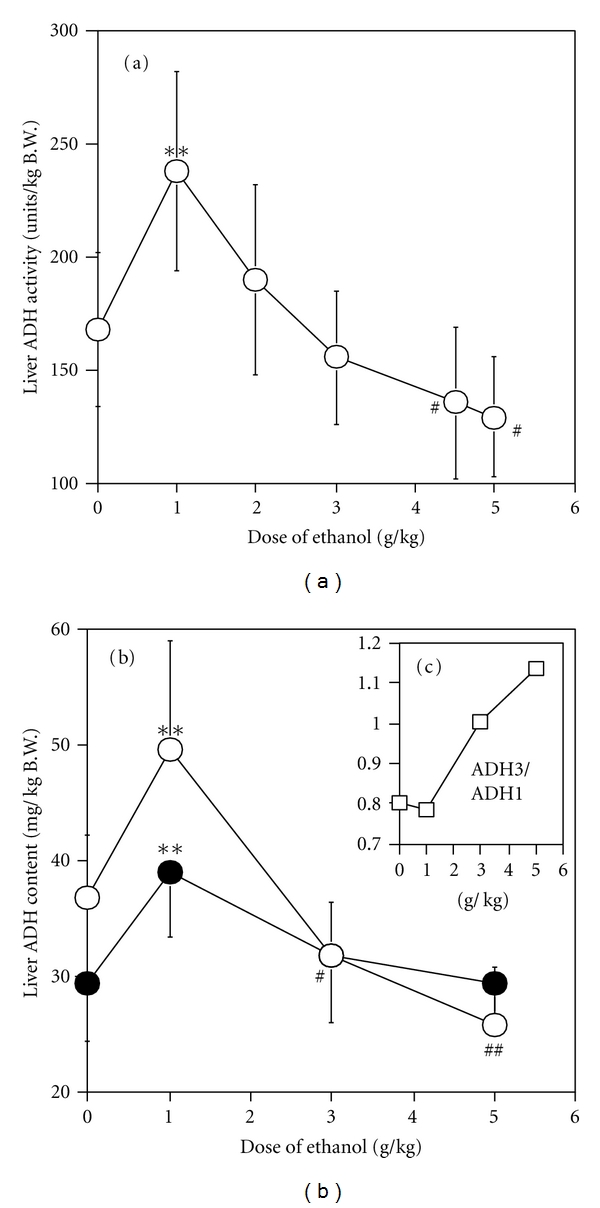
(a) Effect of ethanol dose on liver ADH activity. Three mice were sacrificed at scheduled times during ethanol metabolism after various doses of ethanol: 0.5, 1, and 2 h for 1 and 2 g/kg (9 mice in each dose); 0.5, 1, 2, 4, and 8 h for 3 g/kg (15 mice in the dose); 0.5, 1. 2, 4, 8, and 12 h for 0, 4.5, and 5.0 g/kg (18 mice in each dose), and livers were then removed to prepare liver extracts. The liver ADH activity was measured by the conventional assay with 15 mM ethanol as a substrate at pH 10.7 using liver extracts and is expressed in terms of liver weight/kg body weight. The activities were averaged in each group of ethanol dose to obtain the mean ± SD. (b) Effect of ethanol dose on ADH 1 (○) and ADH 3 (*⚫*) content of liver. In addition to liver ADH activity, the liver extracts were used to measure ADH isozyme contents by EIA using isozyme-specific antibodies. Liver ADH isozyme contents were also averaged in each group of ethanol dose to obtain the mean ± SD. (c) Effect of ethanol dose on ratio of ADH 3 content to ADH 1 content.

**Figure 4:**
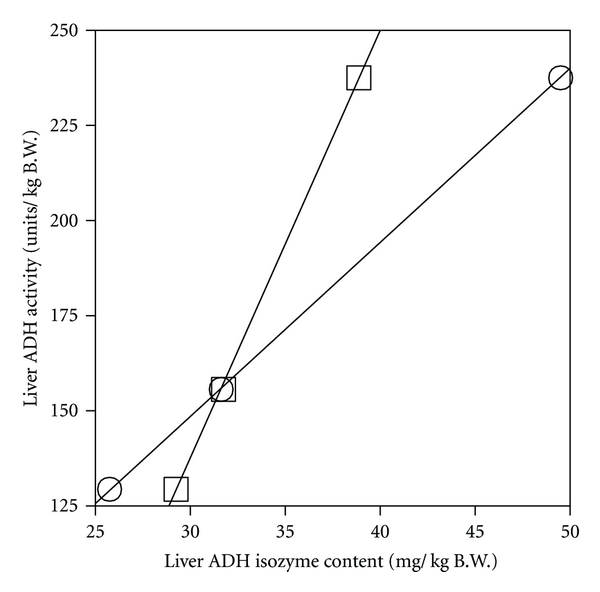
Correlation of liver ADH activity with ADH 1 (○) and ADH 3 (□) contents of liver. Each plot represents the value obtained from Figures 3(a) and 3(b).

**Figure 5:**
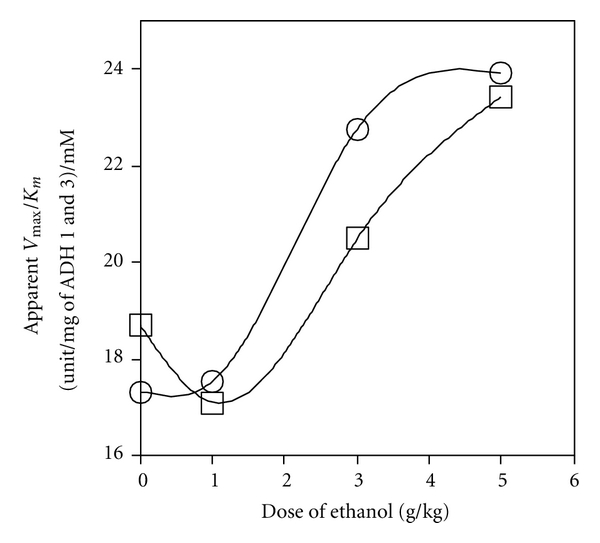
Effect of ethanol dose on catalytic efficiency (*V*_max⁡_/*K*_*m*_) of liver ADH activity. The apparent *V*_max⁡_ and *K*_*m*_ of liver ADH activity were measured using liver extracts from mice 1 h (○) and 4 h (□) after the administration of each dose of ethanol. *V*_max⁡_ is expressed per mg of the sum of the ADH 1 and ADH 3 contents. Each plot represents the average value of 3 mice.

Liver ADH activity (the average over the ethanol-metabolizing time for each ethanol dose) was higher for the 1 g/kg dose (*P* < 0.001), but lower for doses above 2 g/kg (*P* < 0.005 for 4.5 and 5 g/kg) than that of the control (Figure 3(a)). Liver ADH 1 content (the average over the ethanol-metabolizing time) increased for the 1 g/kg dose (*P* < 0.0001) but decreased at higher doses (*P* < 0.05 for 3 g/kg, *P* < 0.0001 for 5 g/kg). Liver ADH 3 content (the average over the ethanol-metabolizing time) also increased for the 1 g/kg dose (*P* < 0.0001) and showed no significant decrease at higher doses (Figure 3(b)). Within ethanol groups, liver ADH activity and liver ADH 1 content decreased dose-dependently (Figures 3(a) and 3(b)), while the ratio of ADH 3 content to ADH 1 content increased dose-dependently (Figure 3(c)). Both the ADH 1 and ADH 3 contents correlated linearly with liver ADH activity (*r* ^2^ = 1.000 for each) (Figure 4). The *V*_max_/*K*_*m*_ of ADH activity of liver extract increased dose-dependently, when measured at 1 or 4 h after administration of ethanol (Figure 5).

### 3.3. Correlation Between Liver ADH Parameters and Pharmacokinetic Parameters

**Figure 6:**
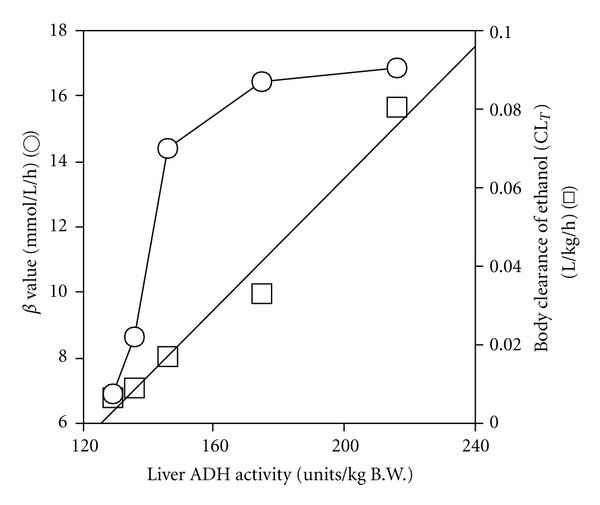
Correlation of *β* and body clearance (CL_*T*_) with liver ADH activity. *β* value (○) was from Figure 2. CL_*T*_ value (□) was the reciprocal of the normalized AUC in Figure 2. Liver ADH activity was from Figure 3(a).

**Figure 7:**
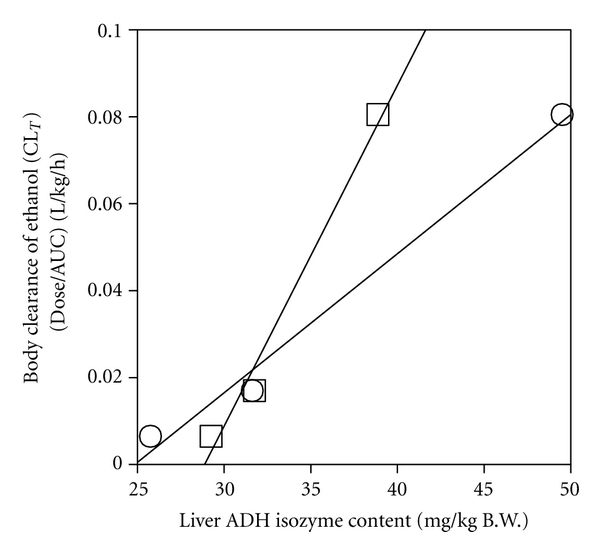
Correlation of body clearance (CL_*T*_) with liver ADH 1 and ADH 3 contents. CL_*T*_ value was from Figure 6. Liver ADH 1 (○) and ADH 3 (□) contents were from Figure 3(b).

Although *β* showed a convex correlation with liver ADH activity, the **C** **L**_**T**_ showed a linear correlation with that activity (**r** ^2^ = 0.972) (Figure 6), and with both liver ADH 1 and ADH 3 contents (**r** ^2^ = 0.988 and 0.987, resp.) (Figure 7).

### 3.4. Two-ADH-Complex Model of Liver ADH Activity

**Figure 8:**
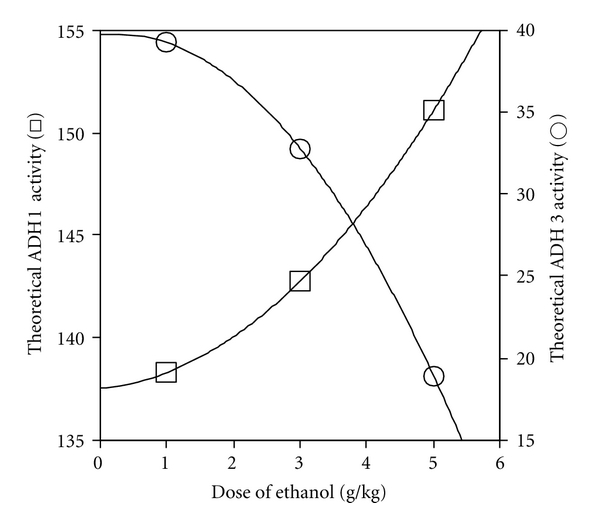
Effect of ethanol dose on theoretical liver ADH 1 and ADH 3 activities in two-ADH-complex model. Liver ADH 1 activity was estimated by multiplying the ADH 1 content by the *V*_max⁡_/mg of ADH 1 (4.0 units/mg). The ADH 3 activity was calculated by subtracting the ADH 1 activity from the total liver ADH activity. The total liver ADH activity was from Figure 3(a) and liver ADH 1 content from Figure 3(b). The theoretical ADH 1 (○) and ADH 3 (□) activities were obtained by normalizing by the ratio of the total ADH activity to that for the control.

**Figure 9:**
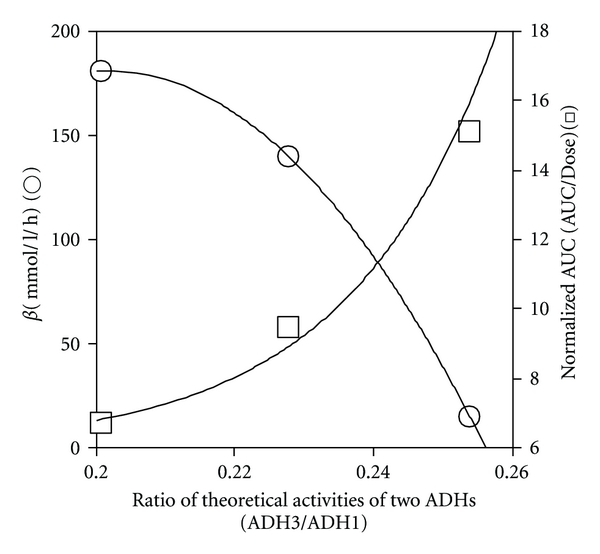
Correlation of *β* and normalized AUC (AUC/dose) of blood ethanol with theoretical ratio of activities of the two ADHs (ADH 3/ADH 1). The values of *β* (○) and normalized AUC (□) were from Figure 2. Theoretical activities of ADH 1 and ADH 3 were from Figure 8.

Analysis of the data based on the two-ADH-complex model of liver ADH activity revealed that the theoretical ADH 1 activity in the liver decreased dose-dependently, whereas the theoretical ADH 3 activity increased dose-dependently (*r* ^2^ = 1.000 for each) (Figure 8). As shown in Figure 9, the increase in the ratio of theoretical activities of ADH 3 to ADH 1 correlated positively with the normalized AUC (*r* ^2^ = 1.000), but negatively with *β* (*r* ^2^ = 0.984).

## 4. Discussion

The elimination rate of alcohol from the blood (*β*) is usually assumed to be constant regardless of the blood ethanol level and to correspond to the rate constant of zero-order or the *V*_max⁡_ of single Michaelis-Menten (M-M) elimination kinetics [7–9]. However, the present study in mice showed that *β* decreased dose-dependently at higher doses (3–5 g/kg) (Figure 2(a)), which was accompanied by a decrease in liver ADH activity (Figure 3(a)). *β* was found to be constant only when liver ADH activity was sufficiently high at low doses of ethanol (1 and 2 g/kg), in which case the liver ADH activity was greater than that of the control. These results mean that, as the ethanol dose increases, the elimination kinetics of ethanol in mice changes from M-M to other kinetics, which involves the decrease of liver ADH activity. Similar results have been reported for rats; *β* or the clearance rate decreased dose-dependently at doses above 2 g/kg, accompanied by dose-dependent decreases of liver ADH activity [27, 28].

AUC, which represents the total amount of ethanol involved in systemic exposure, is an important pharmacokinetic parameter on the bioavailability or toxicity of ethanol. In the present study, the normalized AUC (AUC/dose) showed a concave increase against ethanol dose (Figure 2(a)), probably due to the decrease of liver ADH activity at higher doses of ethanol (Figure 3(a)). Therefore, it showed a linear correlation with the square of the dose, but not with dose itself (see Section 3). These data also indicate that over a wide range of doses the ethanol pharmacokinetics in mice does not simply follow zero-order [7] or M-M kinetics [9], in which the relation between the normalized AUC and ethanol dose shows a proportional correlation.

 Several studies have suggested that the elimination of blood ethanol involves first-order kinetics. In humans [29] and rabbits [23], *β* gradually increased, even at doses of 2 or 3 g/kg, even though the concentration of blood ethanol exceeded that at which the activity of ADH 1, the key metabolic enzyme, is saturated [10, 24]. This type of elimination of blood ethanol is probably due to the participation in ethanol metabolism of higher *K*_*m*_ enzyme(s) without a decrease of liver ADH activity. Fujimiya et al. [23] have proposed a parallel first-order and M-M kinetics for this type of ethanol elimination, in which the relation between the normalized AUC and ethanol dose is also linearly proportional. However, our present results for mice suggest that, just as in humans and rabbits, *β* decreases at higher doses of ethanol than 3 g/kg due to a decrease in liver ADH activity.

The first-order kinetics in alcohol elimination from the blood has been clearly observed in highly intoxicated men with several hundred mM of blood ethanol [20, 21]. ADH^−^ deer mice, which have a low liver ADH activity due to genetically lacking ADH 1 [6], also eliminated blood ethanol following kinetics similar to first-order one up to an ethanol dose of 6 g/kg, at which the maximum blood ethanol concentration reached around 130 mM [30]. These cases of ethanol elimination are probably carried out by a very high-*K*_*m*_ enzyme rather than the key enzyme of ADH 1. 

As non-ADH 1 pathways, MEOS and catalase have been assumed to participate in ethanol metabolism when the blood ethanol level is high because their *K*_*m*_s for ethanol is higher than that of ADH 1 [16, 31–33]. However, neither of these enzymes can explain the first-order kinetics observed at such high levels of blood ethanol in humans and ADH^−^ deer mice because their activities saturate around 50 mM of ethanol [34, 35]. Moreover, any contributions of these two enzymes to systemic alcohol metabolism have not been demonstrated even by using CYP2E1-null or acatalasemic mouse, which genetically lacks MEOS or catalase activity, respectively [17–19]. On the other hand, ADH 4, which mainly localizes in the stomach and also has a higher *K*_*m*_ for ethanol than ADH 1 [36], may play an important role in first-pass metabolism (FPM) to lower BAC and AUC [37]. However, the effect of FPM on BAC is distinct only at low doses of ethanol, which becomes unclear at 2 g/kg and more [37, 38]. In addition, ethanol was injected to mice intraperitoneally in our study. Therefore, the contribution of ADH 4 to BAC and *β* value may be negligible in this study. 

We have recently proposed the participation of ADH 3, which has a very high *K*_*m*_ for ethanol, as a non-ADH 1 pathway of ethanol metabolism. Experiments on ADH 3^−/−^ mice showed that ADH 3 dose-dependently contributed to the elimination of blood ethanol, probably through first-order kinetics [14]. We focused on liver ADH activity and two ADH isozymes, ADH 1 and ADH 3, to analyze elimination kinetics of blood alcohol because the total ADH activity of the liver is closely correlated with the elimination rate of blood alcohol [1–3] and both ADH isozymes have been demonstrated *in vivo* to contribute to alcohol metabolism [13, 14]. 

Although *β* does not always correlate with total liver ADH activity when the activity is excessive [39, Figure 6], body clearance (CL_*T*_) exhibited a linear correlation with liver ADH activity (Figure 6). CL_*T*_, which is the reciprocal of the normalized AUC, is an important parameter indicating the ethanol elimination capacity of the whole body. Many studies have demonstrated that the rate of ethanol elimination in the whole body (CL_*T*_ or *μ*moles/h/animal) correlates with the total liver ADH activity [1, 2, 28, 40]. However, the ethanol elimination in the body cannot be explained solely by ADH 1 [6, 13, 14]. The present study showed that CL_*T*_, which correlated with liver ADH activity (Figure 6), also correlated with both contents of ADH 1 and ADH 3 (Figures 4 and 7). Therefore, it is considered that the capacity to eliminate ethanol from the whole body involves not only ADH 1 but also ADH 3, depending primarily on the level of total liver ADH activity [3].

In the two-ADH-complex model, which ascribes liver ADH activity to both ADH 1 and ADH 3, the theoretical ADH 1 activity decreased dose-dependently (Figure 8), which is experimentally supported by the dose-dependent decrease in liver ADH 1 content (Figure 3(b)). On the other hand, the theoretical ADH 3 activity increased dose-dependently (Figure 8). This is supported by the dose-dependent increase in the apparent *V*_max⁡_/*K*_*m*_ of ADH activity of liver extract, which is expressed in units/mg of the sum of the ADH 1 and ADH 3 contents (Figure 5). The kinetic activation of liver ADH 3 at large doses of ethanol (3–5 g/kg) was also suggested by our previous study [3]. In addition, the theoretical ADH 3 activity also correlated with the ratio of the ADH 3 to the ADH 1 content, which increased dose-dependently (Figure 3(c)). All these experimental data support the idea that the activity of ADH 3 increases dose-dependently due to changes in its content and/or enzyme kinetics in the liver.

The changes in *β* and the normalized AUC against ethanol dose, which showed an inverse linear correlation (Figure 2(b)), may be ascribed to the changes in ADH 1 and ADH 3 activities in the liver (Figure 9). Theoretical ADH 3 activity and normalized AUC show similar dose-dependent increases, whereas theoretical ADH 1 activity and **β** show similar dose-dependent decreases (Figures 2(a) and 8). The hypothesis that the increase in ADH 3 activity accompanying the decrease in ADH 1 activity in the liver increases the normalized AUC and decreases *β* (Figure 9) is supported by the fact that the ethanol-oxidizing efficiency of ADH 3 is much less than that of ADH 1 due to its low affinity for ethanol. Thus, the two-ADH-complex model of liver ADH activity explains well the dose-dependent changes in the pharmacokinetic parameters in mice. The greater participation of ADH 3 and the smaller participation of ADH 1 into ethanol metabolism increase AUC, which in turn raises the ratio of ADH 3 activity to ADH 1 activity (Figure 9). This interdependent increase in the activity ratio and AUC may elevate the bioavailability or toxicity of ethanol. This dynamic theory of the elimination kinetics of ethanol based on the two-ADH-complex model seems to be applicable to alcoholism; regarding patients with alcoholic liver disease, we already reported that the ADH 3 activity increased but the ADH 1 activity decreased with an increase in alcohol intake. Furthermore, the ratio of ADH 3 to ADH 1 activity is significantly related to the incidence of alcoholic cirrhosis of the liver [41].

## 5. Conclusion

The present study suggests that the elimination kinetics of ethanol in mice changes dose-dependently from M-M kinetics to first-order kinetics due to a shift of the dominant metabolizing enzyme from low-*K*_*m*_ ADH 1 to very high-*K*_*m*_ ADH 3. Such a change in the enzymatic pathway of ethanol metabolism may elevate the toxicity of ethanol by nonlinearly increasing AUC due to a decrease in liver ADH activity and sustaining the metabolism through an increase in ADH 3 activity. Thus, ADH 1 and ADH 3, which distribute mainly in parenchymal cells and in sinusoidal endothelial cells of the liver, respectively, seem to regulate pathological effects of alcohol by sharing alcohol metabolism, depending on their catalytic efficiencies, intralobular locations, and responsive potentials to ethanol dose.
